# Energy Analysis of Precooling Air Compressor System

**DOI:** 10.3390/e24081035

**Published:** 2022-07-27

**Authors:** Yu Hu, Weiqing Xu, Guanwei Jia, Guangyao Li, Maolin Cai

**Affiliations:** 1 School of Automation Science and Electrical Engineering, Beihang University, Beijing 100191, China; hyfor18@buaa.edu.cn (Y.H.); liguangyao0419@gmail.com (G.L.); caimaolin@buaa.edu.cn (M.C.); 2 Pneumatic and Thermodynamic Energy Storage and Supply Beijing Key Laboratory, Beijing 100191, China; 3 School of Physics & Electronics, Henan University, Kaifeng 475004, China; jiaguanwei@buaa.edu.cn

**Keywords:** air compressor system, energy conservation technology, pre-cooling, pneumatic-electrical ratio

## Abstract

Energy saving is one of the main technique routes for net zero carbon emissions. Air compressor systems take up a large part of energy consumption in the industrial field. A pre-cooling air compressor system was proposed for energy saving by cooling the air before it flows in a compressor. The energy efficiency of the proposed system was analyzed. As additional energy consumption is required for air cooling, the feasibility of the pre-cooling method for energy saving was analyzed. As the efficiency of the pre-cooling air compressor system is mainly influenced by the environment temperature and humidity, applicability of the system in different regions and at different seasons was discussed. A pilot project was performed to verify the technical feasibility and economics of the proposed system. When the precooling temperature of the pilot system was set to 2 °C, the annual pneumatic-electrical ratio of the system can be increased by approximately 2% in several regions of China. This paper shows the pre-cooling air compressor system is feasible for energy saving.

## 1. Introduction

Mankind’s exploration in the field of energy has never stopped with the development and utilization of new energy sources and the innovation of energy-saving technologies. At present, fossil energy is still the main component of the energy market, accounting for approximately 87% [1]. The large-scale use of fossil energy is accompanied by greenhouse gas emissions, which can cause serious environmental pollution and ecological damage problems, such as acid rain, glacier melt, and rising sea levels. In 2014, the Intergovernmental Panel on Climate Change (IPCC) released the fifth climate change assessment report, which said that since the end of the 19th century, the global average temperature has increased by 0.8 °C [2]. According to the current greenhouse gas emission rate, by the end of this century, the global average temperature rise will reach 4 °C [3]. Improving energy efficiency and reducing carbon emissions are of great significance to the living environment. The industrial field, as an important part of global energy consumption, has always been the key research object for energy conservation and emission reduction.

**Figure 1:**
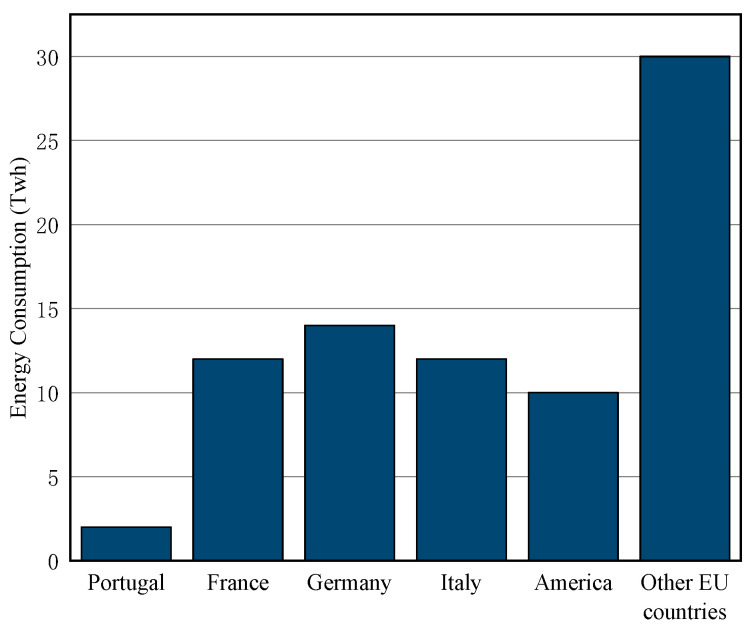
Energy consumption of compressed air systems in some EU countries.

As one of the three major energy transmission systems in the industrial field, pneumatic systems account for a large proportion of industrial energy consumption, and their overall energy efficiency is low [4], which is 20–30% lower than that of electric transmission systems and hydraulic transmission systems. Pneumatic systems waste a lot of energy resources and have great energy-saving potential. At the same time, pneumatic systems are widely used in industry, including food processing, textiles, clothing, paper making, petrochemicals, mechanical mining, wind energy generation, transportation, and buildings [5,6,7,8,9,10]. With the rapid development of industry, the demand for compressed air is increasing year by year. In Australia and the European Union, the energy consumption of the pneumatic system accounts for 10% of the total industrial energy consumption [11]. Figure 1 shows the compressed air energy usage of 15 EU countries [12].

The air compressor system is an important part of the pneumatic system, and its energy consumption accounts for 30–40%. The annual average compound growth rate of the air compressor industry market in China is 4% and that of European countries is 1–2%, which shows that its market scale is expanding rapidly [11]. Therefore, exploring the effective energy conservation technology of air compressor systems is considered to be an important way to improve the efficiency of pneumatic systems and reduce industrial carbon emissions.

Air compressor system energy conservation can usually be considered from the following aspects: first, develop new energy technology and reduce the proportion of the fossil energy market; second, adopt appropriate and feasible energy-saving technologies to reduce carbon emissions in the process of energy use. Finally, carbon capture and storage (CCS) should be promoted to significantly reduce carbon dioxide emissions while meeting the growing global energy demand [13].

The utilization of new energy, such as solar energy, biomass energy, and nuclear energy, can reduce the generation of CO_2_, but because of its intermittence, special requirements for geographical location, and environmental costs, it can only be a useful supplement to fossil energy rather than a large-scale replacement. Fossil energy will remain the foundation of human survival and development far into the future [14]. CCS research focuses on reducing the cost of carbon capture and ensuring the safe and long-term storage of CO_2_ by finding suitable geological storage conditions. Its technology maturity needs further improvement to expand its applicability. Therefore, the study of energy conservation technology is very important for reducing carbon emissions.

At present, air compressor energy-saving technology can be divided into two stages: pre-measures and post-compression treatment. Common pre-measures include component selection (compressor, transmission system, heat exchanger, etc.) and equipment maintenance (leakage, lubrication, etc.). After compression, the energy utilization rate of the air compressor system can be improved by recycling the compression heat to achieve energy savings. In addition, some scholars have performed much research on the compression process. Isothermal compression instead of traditional adiabatic compression can reduce the energy consumption in the compression process. The basic principle of isothermal compression is to strengthen the heat transfer of compressed air and ensure the basic stability of temperature in the compression process. There are two common implementation methods. One method is to use a new liquid piston to directly compress the gas through a liquid column in a fixed volume compression chamber. Experiments [15,16,17] show that compared with the traditional reciprocating piston, the liquid piston can improve the heat transfer area, adapt to different shapes of compression chambers, avoid air leakage and reduce friction resistance [18]. However, at the same time, the increase in the hydraulic system will lead to the problem of gas–liquid mixing, accompanied by noise and vibration. The other way is to inject the liquid spray or foam into the compression chamber to increase the heat transfer area and achieve isothermal performance. This was first proposed by Coney [19]. By spraying, the compression work can be reduced by 28% [20], but the relationship between the additional energy consumption of spray or foam and the size of the compression work needs to be weighed.

In view of the aforesaid problem, it is necessary to explore new and appropriate energy-saving technologies for air compressor systems. The research shows that the energy consumption of the air compressor system is closely related to the suction temperature of *T_in_* and the temperature in the compression process. According to the physical properties of air, the air per unit mass has a higher density and lower water vapor content when the temperature is low. Therefore, when compressing air of the same quality, the lower *T_in_*, the less the energy consumption in the compression process. However, it is worth noting that too low of a *T_in_* will cause the steam to dew under high pressure and form liquid water, which will affect the lubrication effect of lubricating oil and reduce the service life of the air compressor. Also, the increased energy consumption from reducing the *T_in_* of the air compressor must be considered.

**Figure 2:**
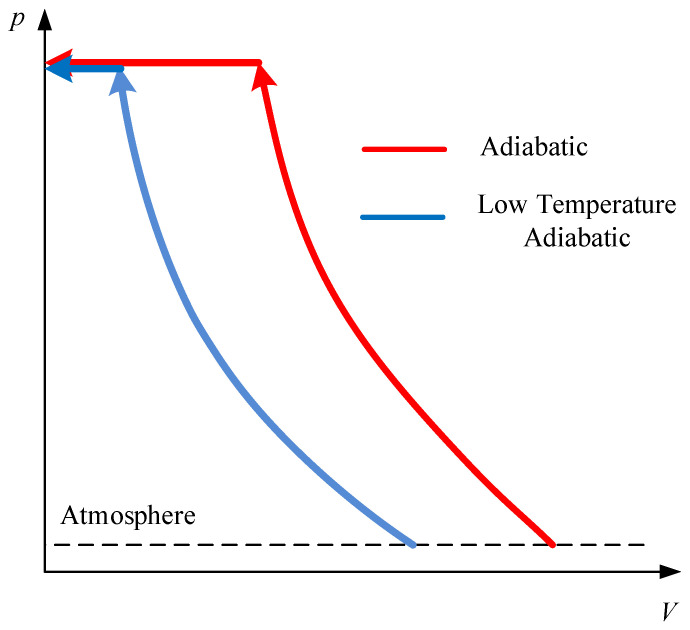
PV diagram of different compression processes.

This paper proposes a pre-cooling air compressor system where the temperature of air is reduced compared with a traditional compressor. When the suction temperature is reduced, the *P-V* curve moves to the left side as the blue curve in Figure 2. As the area enclosed by the *P-V* curve and the coordinate axis decreases, the compression energy consumption decreases. As additional energy consumption is required for air cooling, the feasibility of the pre-cooling method for energy saving is not clear. This paper explores whether the precooling method is feasible for air compressor systems in different regions and seasons.

## 2. Basic Introduction of the Precooling Air Compressor System

### 2.1. Precooling Air Compressor System

As shown in Figure 2, the temperature difference during compression will cause a change in energy consumption. Within a certain range, the lower the temperature during compression is, the higher the compression efficiency. The energy conservation technology of the pre-cooling air compressor system proposed in this paper makes the suction temperature *T_in_* lower than the normal temperature and then compresses the air on the premise of avoiding condensation in the compression process. The air temperature increases gradually with the compression process and is higher than the normal temperature at a certain time node. Throughout the whole compression process, to a certain extent, it reduces the heat loss of air compression and improves compression efficiency.

In addition, the reduction in water vapor content in the air further improves the compression efficiency. Guoda [21] shows that when *T_in_* increases, the content of water vapor in the air increases, and the efficiency of the air compressor system decreases. Precooling can dry the air, which is conducive to improving the compression efficiency of the air compressor system.

**Figure 3:**
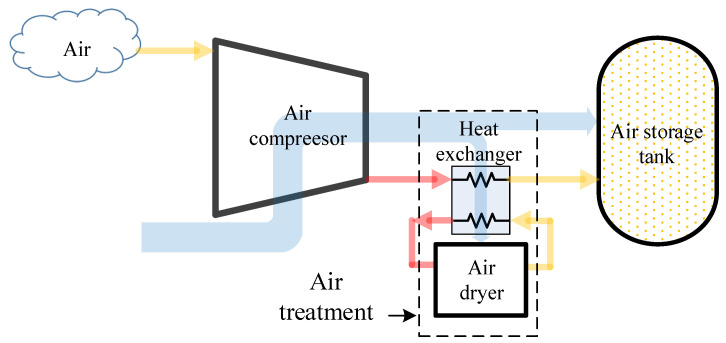
Traditional air compressor system structure.

At present, the traditional air compressor system in the industrial field consists of an air compressor and an air treatment as shown in Figure 3. Air treatment is used to remove the moisture in the high-pressure air for corrosion prevention of equipment in the downstream.

**Figure 4:**
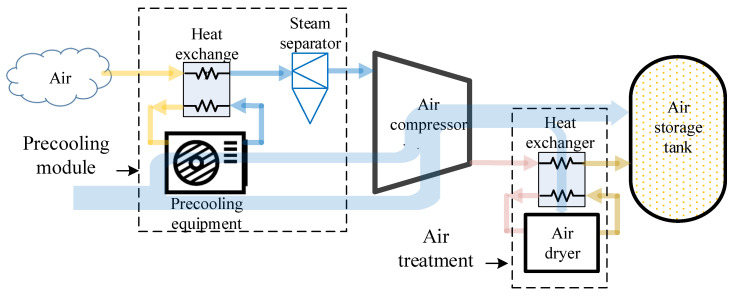
Precooled air compressor system structure.

The pre-cooling air compressor system is proposed to reduce the *T_in_* of the air compressor by adding a precooling module. The water content in low-temperature air is removed by a mechanical steam-water separator. This precooling method is expected to improve the overall efficiency of the air compressor. The system composition is shown in Figure 4.

Compared with the traditional air compressor system, on the one hand, the system increases the precooling energy consumption; on the other hand, the overall efficiency of the air compressor system is improved due to the reduction in *T_in_*. Therefore, a comparative analysis is needed to evaluate the energy-saving effect of the pre-cooling air compressor system.

### 2.2. System Modeling

#### 2.2.1. Traditional Air Compressor Modeling

In the traditional air compressor system, compression work and transmission work are responsible for the total energy consumption of the system. Generally, the compression process belongs to adiabatic compression, and the total energy consumption of the traditional air compressor system is:

(1) $$W_{com}=p_{in}V_{in}\cdot \frac{r^{\frac{k-1}{k}}-1}{\left(k-1\right)/k}/\eta_{com}$$

where *W_com_* is the energy consumption of the air compressor system, *p_in_* is the initial suction pressure at the inlet of the air compressor, *V_in_* is the initial volume of air, *r* is the compressor pressure ratio, and *k* is the adiabatic index, usually 1.4 (take air as the ideal gas), *η_com_* is the isentropic efficiency of the traditional air compressor in practical application [22], usually 0.85.

#### 2.2.2. Precooling Module Modeling

**Figure 5:**
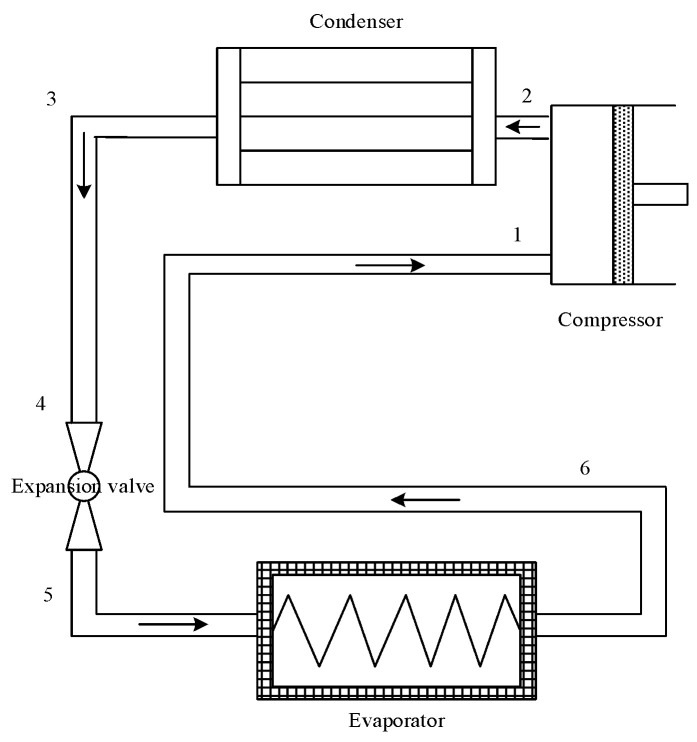
VRC system structure diagram.

The pre-cooling air compressor system proposed in this paper increases the energy consumption of the precooling module based on a traditional air compressor system. The precooling module adopts the common vapor compression refrigeration cycle (VRC), as shown in Figure 5, to establish the model and make the following assumptions:

(1) All components in the precooling module are in a steady state.
(2) The compression processes in the precooling module are reversible and have a given isentropic efficiency.
(3) The throttling processes in the expansion valve are isenthalpic.
(4) The refrigerant at the outlet of the condenser and evaporator is saturated.
(5) The pressure loss and heat loss of the refrigerant in the refrigeration cycle are ignored.

On this basis, the following equations are established according to the conservation theorem of mass and energy. In the VRC system, the energy consumption of compression per unit mass of refrigerant *w_cold_unit_* can be expressed as:

(2) $$w_{cold\_unit}=(h_{2}-h_{1})/\eta_{cold\_com}$$

where *h*_1_ and *h*_2_ are the specific enthalpy of the refrigerant at the inlet and outlet of the compressor, respectively, and *η_cold_com_* is the isentropic compression efficiency of the refrigeration compressor, usually 0.85 [23];

After isobaric condensation, the refrigerant at the condenser outlet is saturated under condensation pressure, and there is a certain subcooling degree. The pressure at the inlet of the expansion valve is constant, and the enthalpy in the throttling process remains unchanged.

(3) $$h_{4}=h_{5}$$

where *h*_4_ and *h*_5_ are the specific enthalpies of the refrigerant at the inlet and outlet of the expansion valve, respectively.

For the evaporator, the refrigerating capacity per unit mass of refrigerant *q_cold_* can be presented by:

(4) $$q_{cold}=h_{6}-h_{5}$$

where *h*_6_ is the specific enthalpy of the refrigerant at the outlet of the evaporator.

Then, the coefficient of performance (*COP*) of the precooling module can be expressed as:

(5) $$COP=q_{cold}/w_{cold\_unit}$$

#### 2.2.3. Exergy Analysis

Exergy analysis of the two air compressor system is discussed in this section. The exergy flow of the systems is investigated to reveal the performance improvement of the proposed system. Without considering the kinetic energy and potential energy, exergy *Ex* of fluids in the system can be expressed as [24]:

(6) $$Ex=m\left[\left(h-h_{0}\right)-T_{en}\left(s-s_{0}\right)\right]$$

where *h* and *s* are specific enthalpy and specific entropy of air, respectively, *h*_0_, *s*_0_ represent the enthalpy and entropy of air at reference temperature *T_en_* (293.15 K) and reference pressure 101.3 kPa.

**Figure 6:**
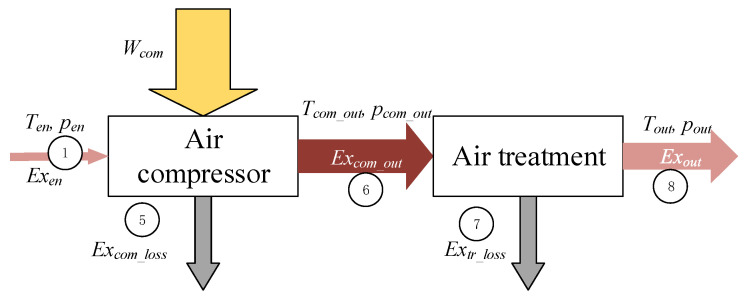
Exergy flow of a traditional air compressor system.

The exergy of air in both the traditional air compressor system and the pre-cooling air compressor system is calculated. In the case of the traditional air compressor system, air under environmental conditions enters the air compressor, discharges from the compressor at high pressure and high temperatures, flows in an air treatment component, and discharges at high pressure, atmosphere temperature. The exergy flow is shown in Figure 6.

*W_com_* of the compressor results in an increase in the exergy in the air. Irreversible of the air compressor results in the exergy loss *Ex_com_loss_* in the air. The exergy of the air in the entrance *Ex_en_* and outlet *Ex_com_out_* of the compressor is written as.

(7) $$W_{com}=\left(Ex_{com\_out}-Ex_{en}\right)+Ex_{com\_loss}$$

where, the subscript *en* represents the environmental condition. The subscript *com* represents compressor.

As the air is cooled in the air treatment component, a portion of the exergy is lost,

(8) $$Ex_{com\_out}=Ex_{out}+Ex_{tr\_loss}$$

where, *Ex_out_* is the exergy out of the whole air compressor system, *Ex_tr_loss_* is the exergy loss of the air treatment.

**Figure 7:**
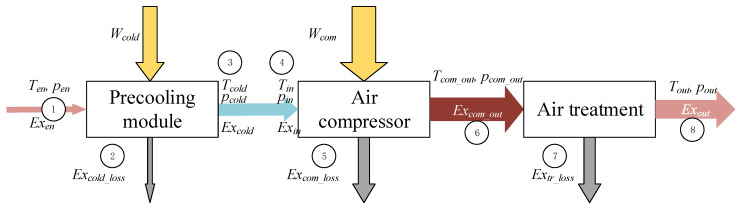
Exergy flow of a pre-cooling air compressor system.

In the case of precooling air compressor system, as shown in Figure 7, air flows through a precooling module and then into the air compressor. The energy consumption of the precooling module *W_cold_* results in an increase in the exergy in the air. Irreversible of the precooling module results in the exergy loss *Ex_cold_loss_* in the air. The exergy of the air in the entrance *Ex_en_* and outlet *Ex_cold_* of the precooling module is written as.

(9) $$W_{cold}=\left(Ex_{cold}-Ex_{en}\right)+Ex_{cold\_loss}$$

where, the subscript *cold* represents the precooling module.

Ignoring the loss between the precooling module and the air compressor, then

(10) $$Ex_{cold}=Ex_{in}$$

where, the subscript *in* represents the inlet of the air compressor.

A typical work condition is considered in this study to compare the exergy loss of the traditional system and the precooling system. Table 1 shows the exergy flow of 1 m^3^ of air in the two systems. In the traditional system, the total exergy consumption is 324.66 kJ. 14.59% of the exergy is lost in the air compressor. 22.59% of the exergy is lost in the air treatment component. In the precooling system, the total exergy consumption is 310.3 kJ. 1.59% of the exergy is lost in the precooling module. 14.44% of the exergy is lost in the air compressor. 18.24% of the exergy is lost in the air treatment component.

After precooling, the unit volume of air (20 °C, 101.3 kPa) is pressurized to the same pressure (*r* = 7.5), and the total energy consumption is reduced from 324.66 kJ to 310.3 kJ. After compression, restore the high-pressure air to normal temperature and generate waste heat after treatment by air treatment. When the same amount of high-pressure air is obtained, the energy consumption decreases by 4.42%.

### 2.3. Energy Efficiency Evaluation Index of the Air Compressor System

For the air compressor system, the overall pneumatic-electrical ratio *δ* of the system is usually used as the energy efficiency index to judge the system performance. The pneumatic-electrical ratio refers to the ratio of compressed air production to the energy consumption of the air compressor system. Its definition is:

(11) $$\delta =V_{0}/W_{com}$$

where *δ* is the pneumatic-electrical ratio of the air compressor system, *V*_0_ is the volume of the actually generated high-pressure air under standard conditions, and *W_com_* refers to the compression energy consumption in the process of generating *V*_0_ volume of high-pressure air.

A higher ratio means that an air compressor system that consumes unit energy can produce more high-pressure gas, and has better system performance. In contrast, the system efficiency is lower.

However, for the proposed precooling air compressor system, the overall energy consumption of the system is increased by the refrigeration energy consumption, so its pneumatic-electrical ratio *δ*′ can be expressed as:

(12) $$\delta^{\prime }=V_{0}/\left(W_{com}+W_{cold}\right)$$

where *W_cold_* refers to the energy consumption of the precooling module in the process of *V_0_* volume of high-pressure air generated by the proposed precooling air pressure system, which is defined as the cooling energy consumption.

(13) $$W_{cold}=m_{ref}\cdot w_{cold\_unit}$$

where, *m_ref_* is the refrigerant mass and *w_cold_unit_* is the energy consumption per unit mass in the refrigeration cycle.

## 3. Theoretical Analysis and Results

The energy efficiency of the precooling air compressor system is analyzed in this section. As the precooling air compressor system consists of the air compressor system and the precooling module, the influences of temperatures on the air compressor system and the precooling module are analyzed respectively. Finally, coupling effects are analyzed.

### 3.1. Effect of Suction Temperature T_in_ on the Air Compressor System

The traditional air compressor system is modeled and simulated according to the relevant formulas in Section 2.2.1. When other conditions are consistent, we adjust *T_in_* of the air compressor and explore the changes in the exhaust temperature, exhaust pressure, unit energy consumption and pneumatic-electrical ratio of the air compressor.

**Figure 8:**
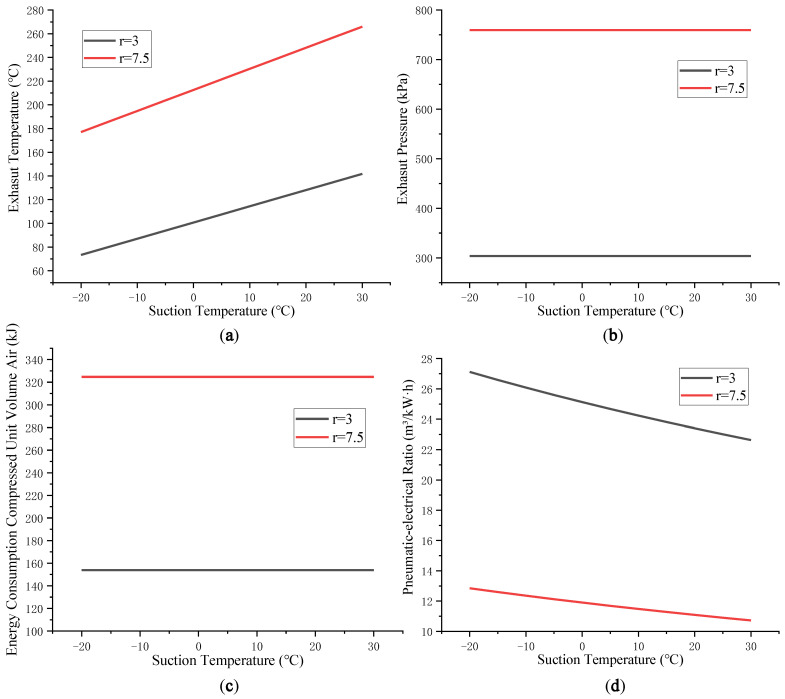
Changes in air compressor system parameters when suction temperature Tin changes: (**a**) Exhaust temperature; (**b**) Exhaust pressure; (**c**) Energy consumption compressed unit volume air; (**d**) Pneumatic-electrical ratio.

The compression process of unit volume air by a traditional air compressor system is taken as the research object. Figure 8 shows the change rule of the exhaust temperature, exhaust pressure, unit energy consumption, and the system energy consumption characteristic parameter pneumatic-electrical ratio when *T_in_* changes.

It can be seen intuitively from Figure 8 that with the increase in *T_in_*, the exhaust temperature of the air compressor system will increase, while the exhaust pressure and the overall energy consumption of the system remain unchanged, and the pneumatic-electrical ratio *δ* shows a downward trend. The reason for this is that when *T_in_* is increased, the air density decreases. When the unit volume of air is compressed, the mass of high-pressure air is reduced, resulting in the reduction of *δ* for the system. When the pressure ratio is low, compressing the gas per unit volume consumes more energy, resulting in the overall pneumatic-electrical ratio of the system being higher than that of the high-pressure ratio. For the air compressor system, reducing *T_in_* can improve the pneumatic-electrical ratio of the system to a certain extent and achieve the optimization effect of energy conservation and emission reduction.

### 3.2. Effect of Temperatures on the Precooling Module

As the precooling module adopts a VRC system, the performance of the precooling module is influenced by the ambient temperature *T_en_* and the refrigeration temperature *T_cold_*. According to formulas in Section 2.2.2, influences of *T_en_* and *T_cold_* on *COP*, *w_cold_unit,_* and *q_cold_* are analyzed.

#### 3.2.1. Impact of Ambient Temperature *T_en_* on *COP*

**Figure 9:**
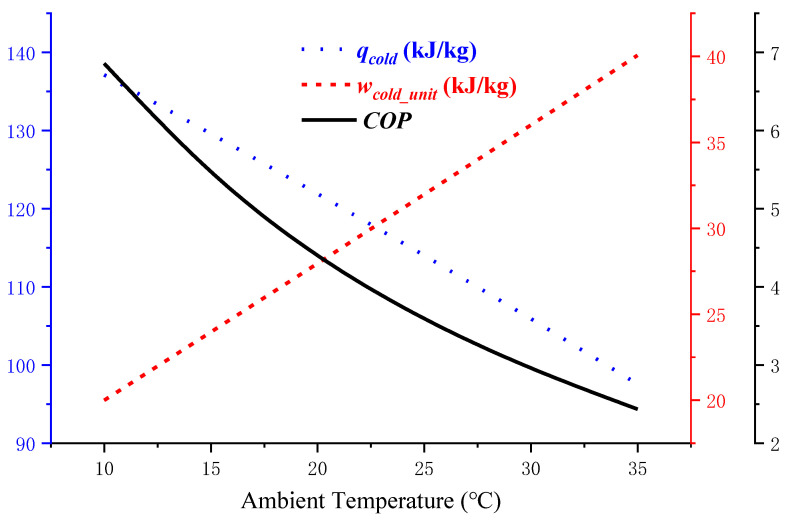
The influence of the ambient temperature *T_en_* on the precooling module.

Generally, in the actual refrigeration demand, the VRC system reduces the higher ambient temperature *T_en_* to the required refrigeration temperature of *T_cold_*. In the model simulation, the common refrigerant R404A is selected, the variation range of *T_en_* is 10 °C to 35 °C, and the required *T_cold_* is 5 °C. According to the empirical parameters, the undercooling degree in the VRC is set to 5 °C, and the superheating degree is set to 8 °C. Under these conditions, the system operating curve for the VRC model is shown in Figure 9.

In Figure 9, the blue dotted line represents the refrigerating capacity that can be transmitted by the unit refrigerant system during the cooling process from *T_en_* to *T_cold_*, while the red dotted line represents the energy consumed by the VRC system; the black curve shows the variation trend of *COP* with *T_en_* under the same *T_cold_*. It can be clearly seen that when *T_cold_* is constant, the *COP* of the VRC system decreases with increasing *T_en_*. This is because the higher *T_en_* is, the higher the corresponding condensation temperature *T_c_* and condensation pressure. Therefore, the energy consumption *w_cold_unit_* consumed by the refrigeration compressor is greater in the VRC system. The higher *T_c_* is, the lower the enthalpy of the saturated liquid refrigerant. Therefore, the cooling capacity *q_cold_* is lower. According to Equation (5), the system *COP* is reduced.

#### 3.2.2. Impact of Refrigeration Temperature *T_cold_* on *COP*

**Figure 10:**
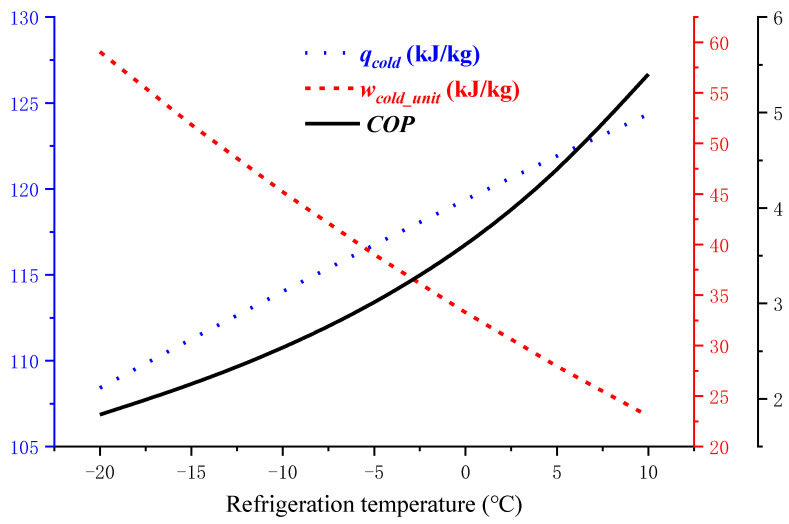
The influence of refrigeration temperature *T_cold_* on the precooling module.

Similarly, the VRC system model can be run for a constant *T_en_*, changing the required *T_cold_*. Setting *T_en_* to 20 °C, and *T_cold_* varies from −20 °C to 10 °C gives the operating curve of the VRC system shown in Figure 10.

Figure 10 shows that the *COP* increases with increasing *T_cold_* under the same *T_en_*. As *T_cold_* increases, the evaporation temperature *T_e_* and the evaporation pressure increase, and *w_cold_unit_*, gradually decreases. During the refrigeration cycle, the higher *T_e_* is, the larger *q_cold_* is. Therefore, the system *COP* increases.

We find that the temperature parameters have a great influence on the performance of the VRC system. It is necessary to choose appropriate temperature parameters according to the actual situation to match the precooling module with the traditional air compressor system to ensure the optimal operation of the new system and maximize energy savings and emission reduction.

### 3.3. Effect of the Operating Temperature T_op_ on the Precooling Air Compressor System

In Section 3.1 and Section 3.2, the influences of temperatures on the air compressor system and the precooling module are analyzed respectively. As the precooling air compressor system consists of the air compressor system and the precooling module, coupling effects are analyzed in this section. As the refrigerator outlet is connected to the air compressor inlet, *T_in_* equals to *T_cold_*. Operating temperature *T_op_* is defined to express *T_in_* and *T_cold_* in the pre-cooling air compressor system.

#### 3.3.1. Difference in the Pneumatic-Electrical Ratio between the Precooling Air Compressor System and the Traditional Air Compressor System

The common compression ratio of the air compressor system is 7.5. The same working conditions were set to compress the unit volume air, *V_in_* = 1 m^3^. For industrial air compressors, the adiabatic index *k* =1.4 (uncertainty analysis is at the end of the article, as shown in Appendix A.2). *T_in_* is set to 20 °C. According to Equations (1) and (11), through simulation calculation, *δ* of the traditional air compressor is 11.084 m^3^/(kW∙h) when *T_in_* is 20 °C.

**Figure 11:**
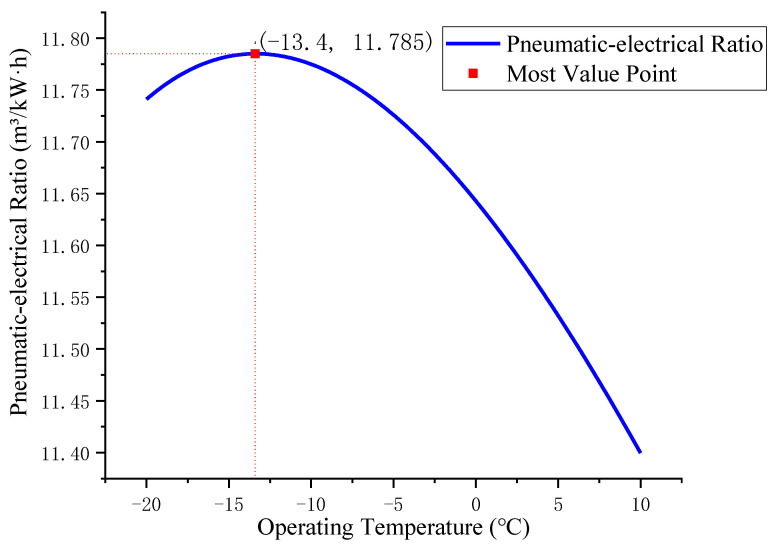
The relationship between the pneumatic-electrical ratio and operating temperature of the system at a single ambient temperature *T_en_*.

R404A is selected as the refrigerant, *T_en_* is set to 20 °C, and *T_cold_* varies from −20 °C to 10 °C. The pre-cooling air compressor system is simulated and calculated. As mentioned in Section 3.1, the lower *T_in_* is, the higher the pneumatic-electrical ratio δ, while the lower *T_cold_* is, the greater *W_cold_* is and the smaller the *COP* is, which is mentioned in Section 3.2.2. Combined with the analysis of Equations (11) and (12), there is an optimal *T_op_* within a certain range, which makes the pneumatic-electrical ratio *δ*′ of the pre-cooling air compressor system reach the best value. According to Equation (12), *δ*′ of the pre-cooling air compressor system, the variation curve of the pneumatic-electrical ratio with respect to *T_in_* can be obtained through simulation calculation, as shown in Figure 11.

It can be seen from the figure that when *T_op_* decreases from 10 °C, *δ*′ gradually increases. When *T_op_* decreases to −13.4 °C, the system reaches the optimal *δ*′ of 11.785 m^3^/(kW∙h) when *T_en_* is 20 °C. Compared with the traditional air compressor system, the pneumatic-electricity ratio is increased by 6.32%.

For the pre-cooling air compressor system, when the unit volume of air has been compressed, with the decrease in *T_op_*, the gas production of the air compressor under standard conditions *V_0_* gradually increases. Although *W_cold_* increases, *δ*′ of the system tends to increase because it accounts for a low proportion of the overall energy consumption of the system; when *T_op_* is further reduced, the proportion of *W_cold_* increases, and the negative effect on *δ*′ is dominant. *δ*′ gradually decreases, so the maximum point appears.

For *T_op_* changes, the proportion change of the energy consumption of the system component is further analyzed, the calculation parameters are shown in Table 2.

**Figure 12:**
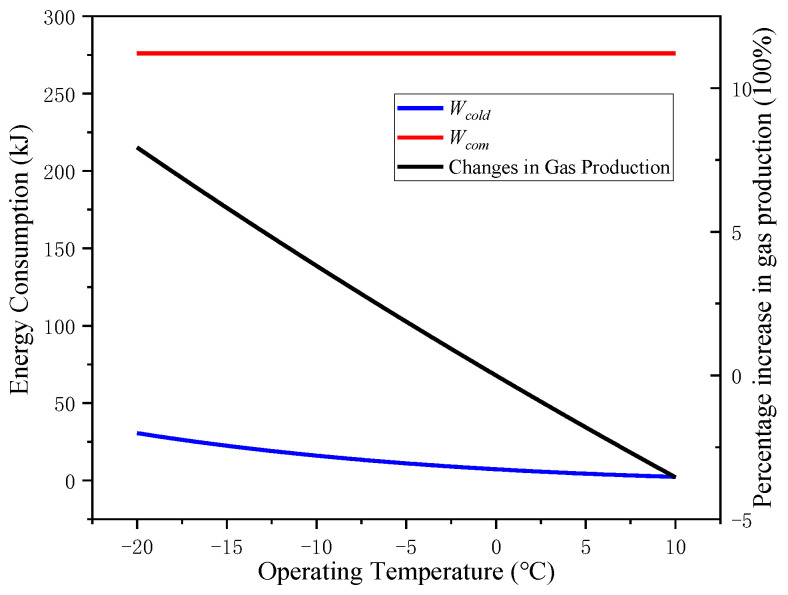
Variation of energy consumption and gas production of system components with operating temperature *T_op_*.

Turning down *T_op_*, the air compressor module’s energy consumption to compress air per unit volume *W_com_* remains unchanged, but the precooling module’s energy consumption *W_cold_* increases, so the overall energy consumption of the system increases gradually. As shown in Figure 12, the black curve shows the effect of *T_op_* on the change percentage of gas production of the system under standard conditions (compared with *T_in_* = 20 °C). Both *W_cold_* and the gas production increase gradually with the decrease in *T_op_*. At first, the increase in gas production is dominant to *δ*′, *δ*′ increases with the decrease in *T_op_*; with the continuous decrease in *T_op_*, the increment of *W_cold_* has more influence on the change in *δ*′, and *δ*′ decreases with the decrease in *T_op_*.

**Figure 13:**
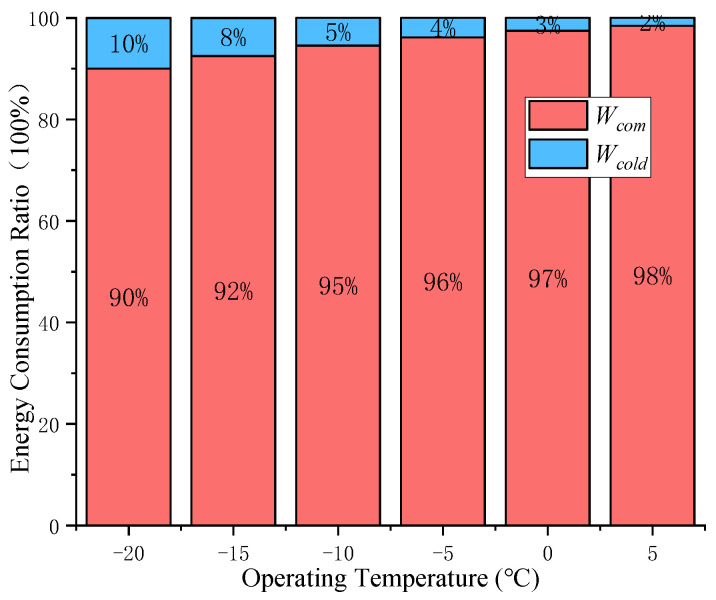
Energy consumption ratio of system components at different operating temperatures.

Within the range of *T_op_* mentioned earlier, several operating temperature points are selected to observe the change trend of the energy consumption ratio of the system, as shown in Figure 13. It can be clearly seen that with the decrease of *T_op_*, the proportion of *W_cold_* in the total energy consumption of the system gradually increases.

#### 3.3.2. Selection of the Optimum *T_op_* for the Precooling Air Compressor System at Different *T_en_*

In the previous section, the relationship between *δ*′ and *T_op_* under a single ambient temperature (*T_en_* = 20 °C) is described. Without considering the influence of other factors, there is an optimal *T_in_* for the pre-cooling air compressor system so that *δ*′ reaches the maximum. However, the ambient temperature *T_en_* will change with the seasons. This section further analyzes the selection of the optimal *T_op_* of the pre-cooling air compressor system under different ambient temperatures.

**Figure 14:**
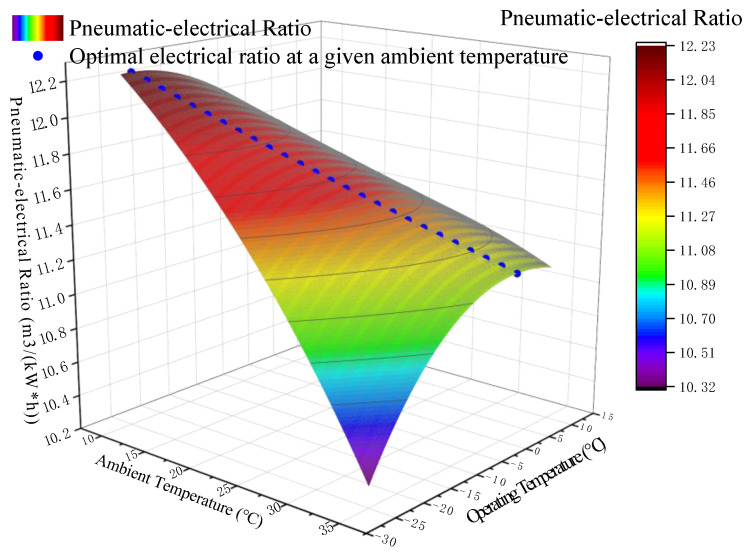
The relationship between the pneumatic-electrical ratio and operating temperature of the system under different ambient temperatures *T_en_*.

*T_en_* is changed in the range of 10 °C to 35 °C, the *T_op_* of the system ranges from −30 °C to 10 °C, and the other conditions are the same as before. The curve diagram shown in Figure 14 is obtained by running the system model. The curved surface in the diagram represents the value of the system δ′ under different *T_en_* and *T_op_*; the blue dot indicates the optimal *T_op_* and the optimal pneumatic-electricity ratio δ′ at a given *T_en_*.

The ambient temperature *T_en_* and the corresponding optimal operating temperature *T_op_* are refined into a two-dimensional diagram, as shown in Figure 14. It can be found that the optimal *T_op_* varies with different *T_en_*, and the two are approximately linear. With the alternation of seasons, it is necessary to adjust the *T_op_* of the system in a timely manner according to the change in *T_en_*. Further analysis shows that the difference between the two values fluctuated around 33 °C. This means that under the set conditions, once *T_en_* is determined, the optimal operating temperature range can be obtained.

**Figure 15:**
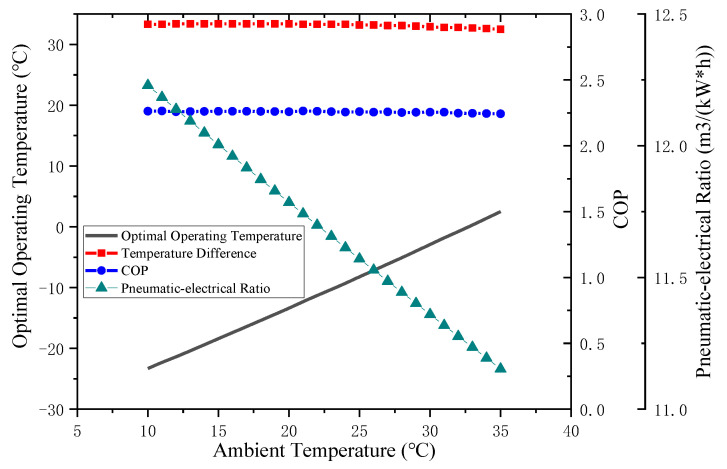
Parameters related to optimal operating conditions at different ambient temperatures.

The correlation between air temperature and density determines that the change in air production of the air compressor system is related to suction temperature *T_in_*. The relationship between the pneumatic-electricity ratio and suction temperature for the traditional air compressor system is shown in Figure 8d. For a specific refrigerant, the energy consumption and *COP* of the VRC system are related to the inlet and outlet temperature difference, that is, to the difference between *T_en_* and *T_cold_*. While the air compressor system compresses the same volume of gas, its energy consumption basically remains unchanged, as shown in Figure 8c. Therefore, after adopting the precooling module, the temperature difference affects the change in the gas production and the energy consumption of the system; that is, the pneumatic-electricity ratio of the system is mainly influenced by the difference between *T_en_* and *T_cold_*. As shown in Figure 15, under the optimal *T_op_* corresponding to the specific *T_en_*, the change in *COP* is small (less than 0.02). The relationship between *T_en_* and optimum *T_op_* can be obtained by traversing a certain temperature range.

Timely adjustment of the *T_op_* for the pre-cooling air compressor system puts forward higher requirements for the precooling module. Currently, frequency conversion refrigeration equipment or multistage refrigeration can be used to achieve this function.

## 4. Seasonal Variation of the System in Different Regions

To analyze the energy conservation effect of the pre-cooling air compressor system more comprehensively, this chapter studies the air compressor systems in different regions.

### 4.1. Operating Condition Difference of the Air Compressor System in Different Regions

**Figure 16:**
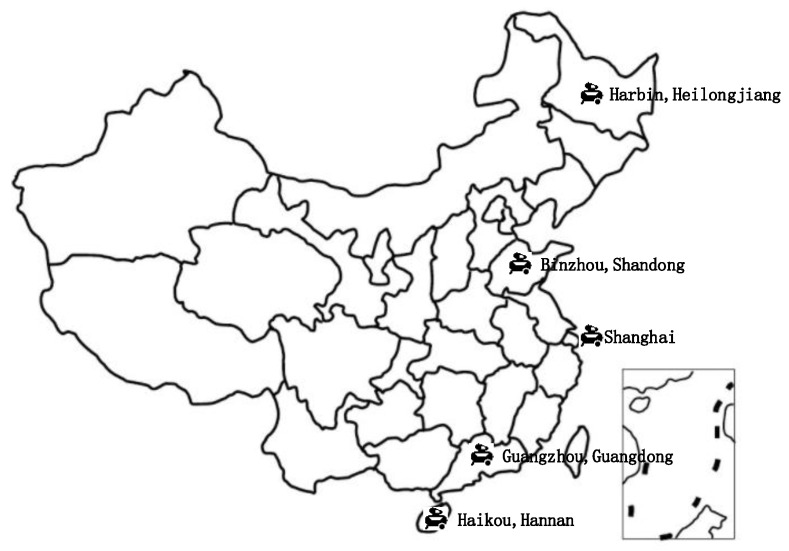
Geographical distribution of the selected Chinese cities where the air compressor system is located.

According to the geographical location of China, five representative cities with climatic characteristics are selected from south to north, namely, Haikou, Hainan; Guangzhou, Guangdong; Shanghai; Binzhou, Shandong; and Harbin, Heilongjiang, as shown in Figure 16.

#### 4.1.1. Traditional Air Compressor System

**Figure 17:**
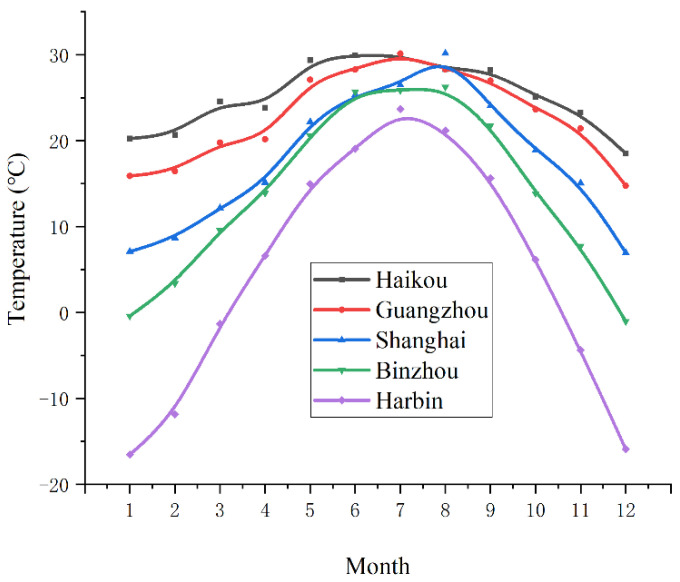
Mean monthly temperature curve for different regions.

As shown in Figure 17, the annual mean monthly temperature change curve in the 5 regions in 2020 is moving down from south to north, and the temperature gradually decreases. The monthly average temperature in Haikou is relatively high, basically maintained at over 20 °C, and the air temperature difference between winter and summer is small; however, in Harbin, the monthly average temperature is low, and the gap between winter and summer is large, and the monthly average temperature difference is as high as 40 °C.

**Figure 18:**
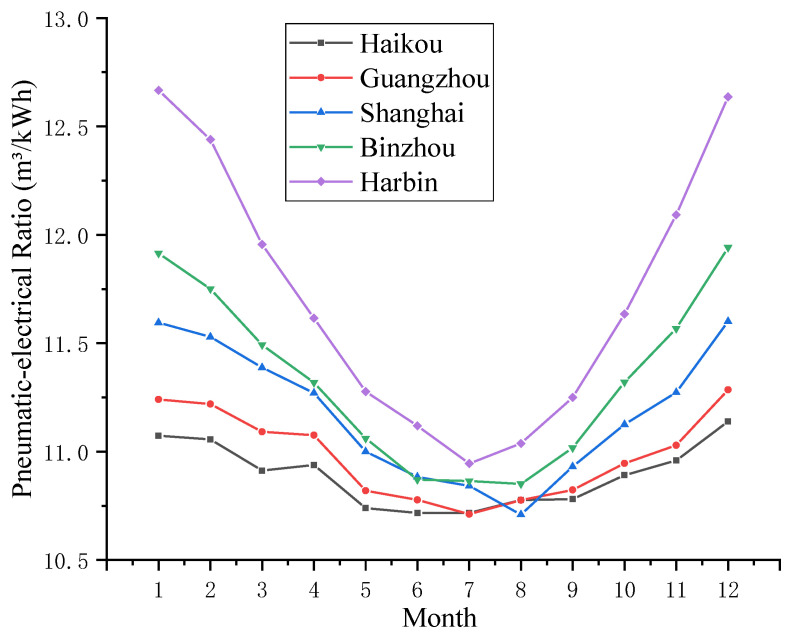
Monthly pneumatic-electricity ratio curve of air compressor systems in different regions.

An air compressor system with the same performance can be run in these five areas, and the monthly pneumatic-electricity ratio curve of air compressor systems in different regions can be obtained, as shown in Figure 18. The variation trend of the pneumatic-electricity ratio is contrary to that of the monthly mean temperature. The main reason is that the physical properties of air are affected by ambient temperature.

#### 4.1.2. Precooling Air Compressor System

According to the above analysis, the precooling module is added to improve the traditional air compressor system. Taking into account the influence of icing conditions on the performance of air compressors, the operating temperature *T_op_* is set to 2 °C. When *T_en_* is greater than *T_op_*, the precooling module works; conversely, it stops working.

**Figure 19:**
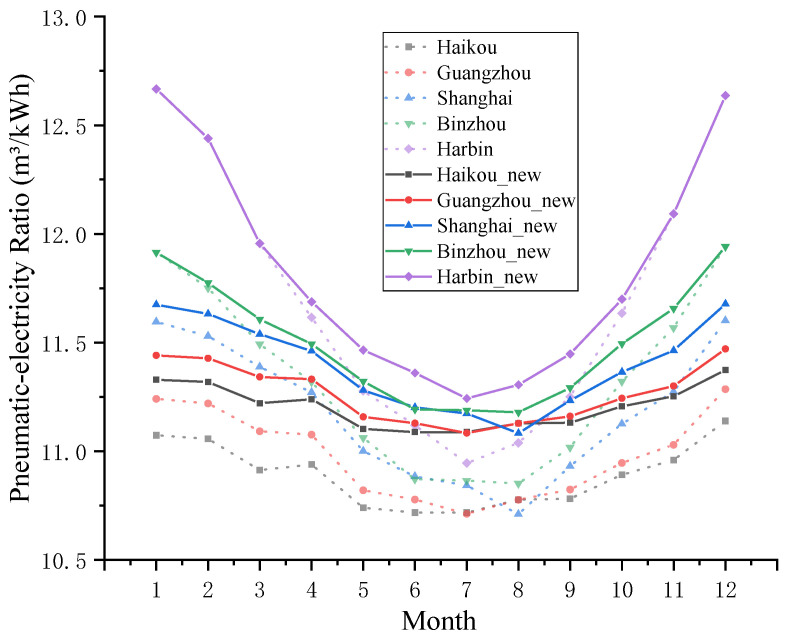
The change in the pneumatic-electrical ratio before and after using the precooling module.

**Figure 20:**
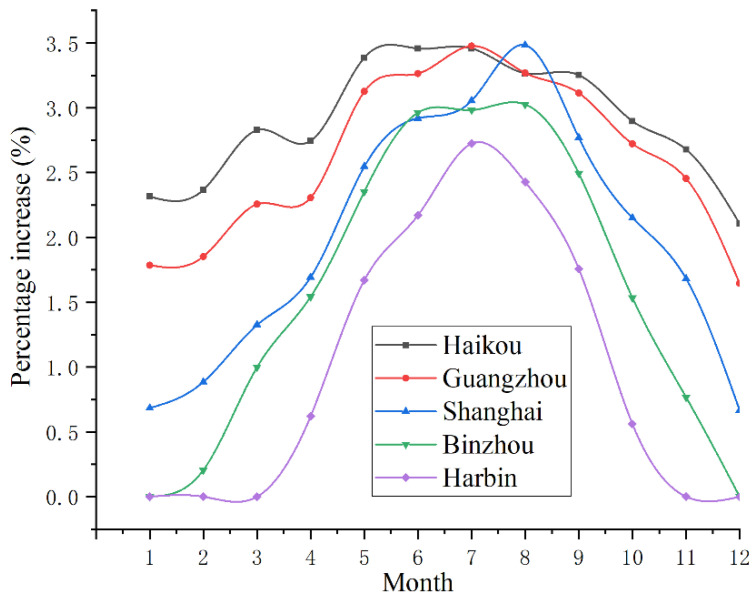
The ratio of change in the pneumatic-electrical ratio before and after using the precooling module.

Assuming that the *COP* of the VRC system is 2, the monthly variation trend of the pneumatic-electricity ratio of the pre-cooling air compressor system in different regions can be obtained, as shown in Figure 19. Among them, the dashed line represents the change in the pneumatic-electricity ratio *δ* of the traditional air compressor system, and the solid line represents the pneumatic-electricity ratio *δ*′ of the new system. Based on the difference in the pneumatic electricity before and after improvement, the percentage increase can be obtained, as shown in Figure 20.

Combined with Figure 19 and Figure 20, the influence of the precooling module on the pneumatic-electricity ratio of the air compressor system is quite different in different regions. In Haikou, the monthly average temperature is located at 20–30 °C, the effect of the precooling module is more obvious, and the pneumatic-electricity ratio can be increased by 2.5–3%. In the two time periods from January to March and November to December, *T_en_* is lower than *T_op_* in Harbin, the precooling module does not work, and the pneumatic-electricity ratio remains unchanged; however, in other months, there is a gap in the improvement of the pneumatic-electricity ratio, which can reach approximately 2.5% in summer (June to August) and only 0.5% in spring and autumn. Starting from Haikou, from south to north, the proportion of the annual pneumatic-electricity ratio shifted down, and the seasonal difference gradually became obvious.

Therefore, when using the precooling module to improve the traditional air compressor system, the refrigeration form and operating parameters of the system should be reasonably selected according to the local conditions and different climatic characteristics of each region.

### 4.2. Operation of the Precooling Air Compressor System in a Region

According to the seasonal variation in climate in some areas, different requirements for traditional air compressor systems and matching precooling modules are proposed. Here, the air compressor system in Binzhou is selected as the research object for analysis.

#### 4.2.1. Differences in the Matching of Precooling Modules in Different Seasons

Section 3.3.2 mentions that the optimal *T_op_* for the pre-cooling air compressor system is different under different *T_en_*. It is necessary to match the appropriate precooling module and adjust the operating temperature in time to ensure that the system reaches the optimal working state.

**Figure 21:**
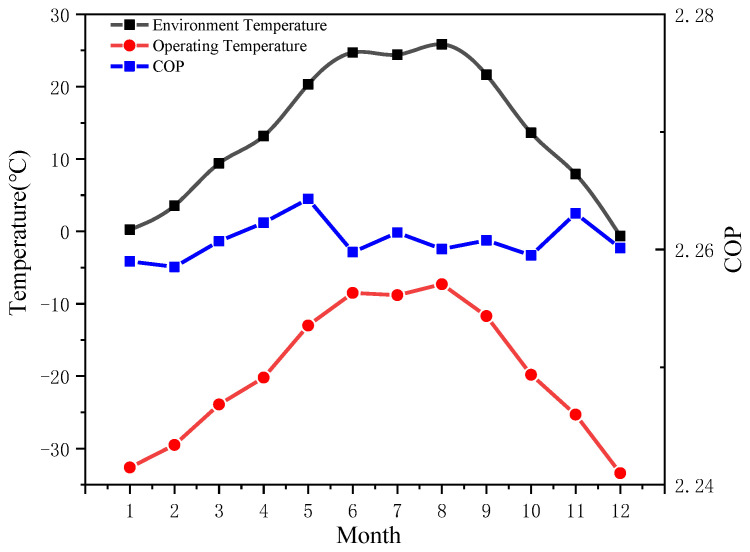
Different monthly average temperatures and the corresponding optimal operating temperatures and system *COP*s.

**Figure 22:**
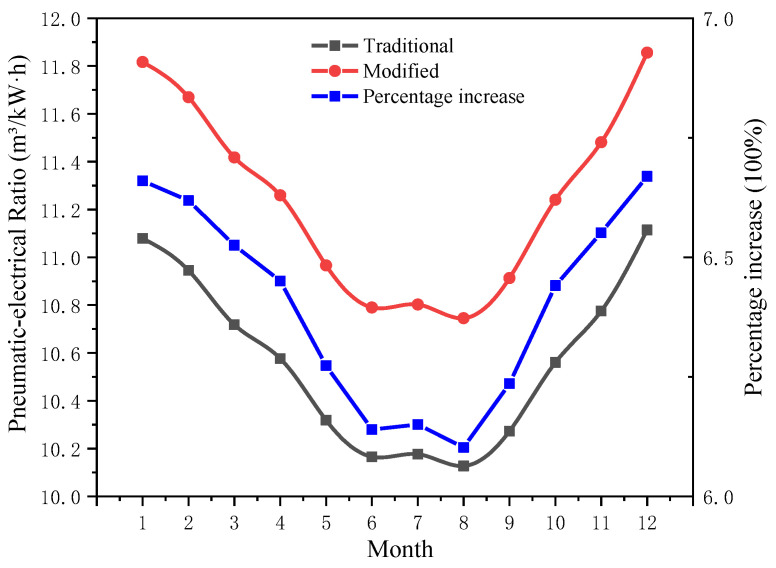
Monthly change trend and percentage increase in the system pneumatic-electrical ratio.

Figure 21 shows the average temperature of Binzhou in different months, as well as the corresponding optimal refrigeration temperature and the *COP* for the precooling module. In Figure 22, the monthly change trend of the pneumatic-electricity ratio of the two air compressor systems and the increased percentage of the pneumatic-electricity ratio are shown. By reasonably reducing the suction temperature, the pneumatic-electricity ratio of the air compressor system can be better improved. In summer (June to August), the average temperature is relatively high, approximately 25 °C, the optimal *T_op_* of the pre-cooling air compressor system is near −8 °C, and the pneumatic-electricity ratio of the system is up to 10.8 m^3^/kW∙h, which is 6.4% higher than the traditional air compressor system (10.15 m^3^/kW∙h); in spring and winter, the regional average ambient temperature is low, and the optimal *T_op_* of the system also decreases. In January and December, *T_en_* is approximately 0 ℃, while the optimal *T_op_* reaches −33 ℃, and the pneumatic-electricity ratio increased to 11.85 m^3^/kW∙h, which is 6.65% higher than that of the traditional air compressor system (11 m^3^/kW∙h). The *COP* of the precooling module is basically stable at 2.26 throughout the year. Currently, refrigeration technology can meet performance requirements.

Results show that when adopting the pre-cooling method to improve the air compressor system, the regional seasonal ambient temperature change should be considered, and the precooling module should be adjusted in real-time to ensure the efficient operation of the system.

#### 4.2.2. Selection of the Precooling Module in Practical Applications

**Figure 23:**
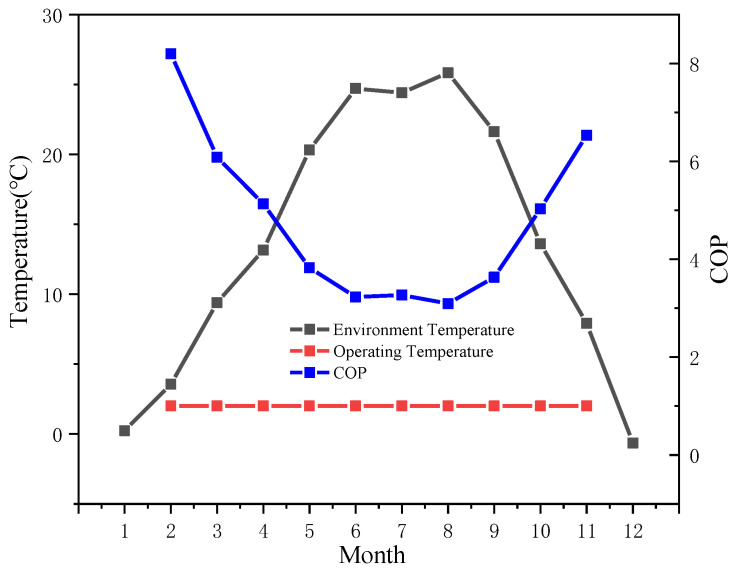
For a fixed operating temperature, the system’s *COP* varies under different ambient temperatures.

**Figure 24:**
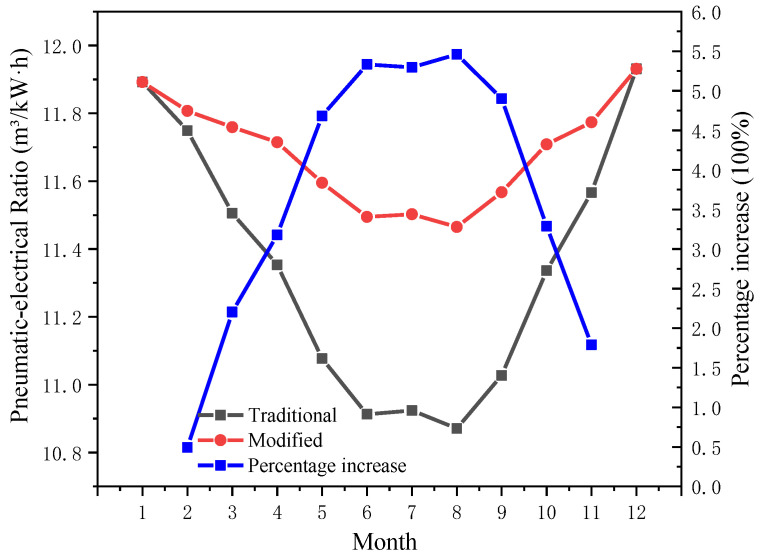
When the operating temperature is set, the change trend and change percentage of the pneumatic-electricity ratio before and after using the precooling module.

In the previous section, when other factors, such as refrigeration difficulty, are not considered, the lower the regional *T_en_* is, the more obvious the pre-cooling method improves the pneumatic-electricity ratio of the traditional air compressor system, and the better the energy-saving effect of the system. In practical applications, an air compressor suction temperature that is too low will lead to icing and affect the normal operation of the system. Combined with the previous analysis, *T_op_* is uniformly set to 2 °C, which is as close to the optimal *T_op_* as possible while avoiding icing. When *T_en_* is lower than 2 °C, the precooling module stops working. Now, the system performance is analyzed to obtain the monthly operating curve of the system at this *T_op_*, as shown in Figure 23 and Figure 24.

As shown in the figure, in January and December, the precooling module stops working. Figure 23 shows that at a given *T_op_*, the higher *T_en_* is, the lower the *COP*, which is consistent with the theoretical analysis in Section 3.2.1; it can be seen from Figure 24 that in the practical application of the system, compared with that of the traditional air compressor system, the higher *T_en_* is, the better the optimization effect of the pneumatic-electricity ratio is. In August, the average temperature was 25.8 °C. After the improvement of the precooling module, the pneumatic-electricity ratio of the system that was 10.8712 m^3^/kW∙h increased to 11.4649 m^3^/kW∙h, up 5.46%; however, in February, the average temperature was 3.56 °C, and the ratio increased by only 0.49%, from 11.7492 m^3^/kW∙h to 11.8072 m^3^/kW∙h.

In practical applications, many problems, such as icing, need to be considered. To ensure an energy-saving effect, multistage refrigeration can be used in the precooling module. According to the climatic characteristics of different seasons, different series of refrigeration can be realized to match the refrigeration demand of the pre-cooling air compressor system for the whole year as much as possible to ensure the efficient and stable operation of the system.

## 5. Conclusions

In this paper, a pre-cooling air compressor system that applies precooling equipment to reduce the suction temperature of the air compressor system was proposed. The pneumatic-electricity ratio (*δ*) of the air compressor system was increased with decreases in the suction temperature. As additional energy consumption is required for air cooling, the feasibility of the precooling air compressor system depends both on the pneumatic-electricity ratio of the air compressor system and *COP* of the precooling module.

Reducing operating temperature *T_op_* results in increases in *δ* of the air compressor system, but decreases in *COP*. The optimal operating temperature *T_op_* was obtained when the pneumatic-electricity ratio *δ*′ of precooling air compressor system achieved maximum. When the ambient temperature *T_en_* was 20 °C, the operating temperature *T_op_* of the proposed system was −13.4 °C; the pneumatic-electricity ratio *δ*′ reached its optimum 11.785 m^3^/kW∙h, which is 6.32% higher than that for the traditional air compressor (11.084 m^3^/kW∙h).

The efficiency of the precooling air compressor system varies with different region and different season, as it is mainly influenced by the environment temperature and humidity. The pneumatic-electricity ratio can be improved in the regions where environment temperature is high and humidity is low, at the seasons when environment temperature is high and humidity is low. Cooling energy consumed by water vapor in air takes up to 2~41% of cooling capacity and it should not be ignored.

A pilot project was performed in Binzhou city, Shan Dong province, China. Considering the limitations of other factors, the reasonable *T_op_* of the system was set to 2 °C, and the higher the average temperature was, the better the system pneumatic-electricity ratio was. At different *T_en_*, the *COP* corresponding to the optimal *T_op_* was greatly different. The higher the average temperature was, the smaller the *COP* of the precooling module. This is instructive to the energy-saving optimization of the traditional air compressor system in industrial applications. If the precooling module adopts the form of multistage refrigeration, it can maximize the matching of the annual refrigeration demand of the system.

There are still many areas to be improved in the follow-up. Firstly, considering the influence of water vapor in the air, the difference of cooling energy consumption under different relative humidity is analyzed, as shown in Appendix A.1. Secondly, the analysis of the economic feasibility of the system in practical application is shown in Appendix A.2. Finally, research on improving the overall energy efficiency of the air pressure system can be carried out from the perspective of waste heat recovery and utilization, please see Appendix A.4.

## Figures and Tables

**Table 1:** Comparison of related parameters.

System		①	②	③	④	⑤	⑥	⑦	⑧
Traditional	*T*/K	293.15		-	*-*	521.32	521.32	293.15	293.15
	*P*/kPa	101.3		-	-	759.75	759.75	759.75	759.75
	*Ex(W)*/kJ	0.00		-	-	47.37	277.30	73.34	203.96
Precooling	*T*/K	293.15	275.15	275.15	275.15	489.31	489.31	293.15	293.15
	*P*/kPa	101.3	101.3	101.3	101.3	759.75	759.75	759.75	759.75
	*Ex(W)*/kJ	0.00	4.94	0.70	0.70	44.80	260.57	56.61	203.96

**Table 2:** Calculation of related parameters when *T_op_* changes.

	*T_op_* (℃)	−20	……	−13.4	……	10	20
Parameter							
*T_in_* (K)		273.15	……	279.75	……	303.15	313.15
*P_in_* (101.3 kPa)		101.3	……	101.3	……	101.3	101.3
*V_in_* (m^3^)		1	……	1	……	1	1
*W_com_* (kJ)		275.96438	……	275.96438	……	275.96438	275.96438
*T_en_* (K)		293.15	……	293.15	……	293.15	293.15
*T_cold_* (K)		273.15	……	279.75	……	303.15	313.15
*W_cold_* (kJ)		30.58789	……	20.23258	……	2.32267	0
*V_0_* (m^3^)		1.07933	……	1.05179	……	0.96457	1
Percentage increase in gas production (%)		7.933	……	5.179	……	−3.543	-

**Figure A1:**
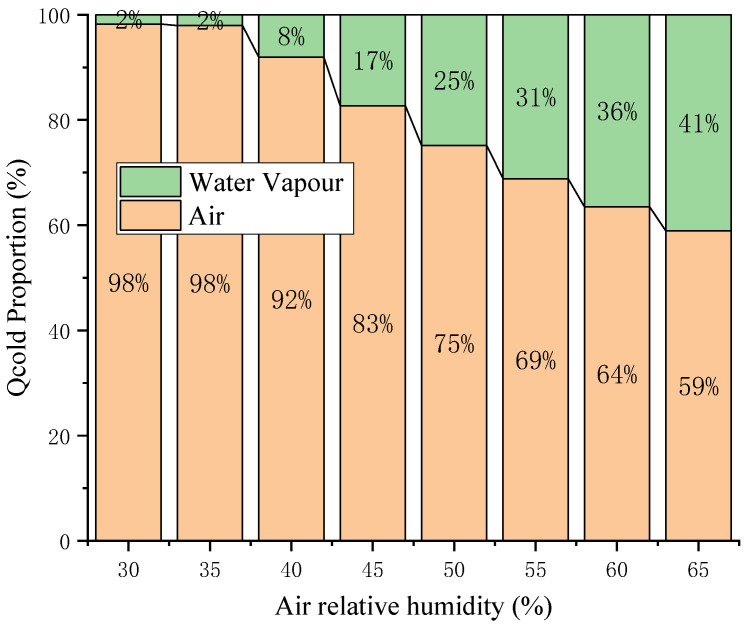
Proportion of air and water vapor cooling energy consumption.

## Nomenclature

*T_in_*	Suction temperature (°C)	*s*_0_	Entropy of air at 293.15 K and 101.3 kPa (kJ/(kg·K))
*W_com_*	Energy consumption of the air compressor system (kJ)	*Ex_com_loss_*	Exergy loss of traditional air compressor (kJ)
*p_in_*	Suction pressure (kPa)	*W_cold_*	Energy consumption of the precooling module (kJ)
*V_in_*	Initial volume of air (m^3^)	*Ex_tr_loss_*	Exergy loss of air treatment (kJ)
*r*	Compressor pressure ratio	*Ex_cold_loss_*	Exergy loss of precooling module (kJ)
*k*	Adiabatic index (1.4)	*δ*	Pneumatic-electrical ratio of the traditional air compressor system (m^3^/kW·h)
*η_com_*	Isentropic efficiency of traditional air compressor (%)	V_0_	Volume of high pressure air under standard condition (m^3^)
*w_cold_unit_*	Energy consumption for compressing unit mass of refrigerant (kJ/kg)	*δ*′	Pneumatic-electrical ratio of the precooling air compressor system (m^3^/kW·h)
*η_cold_com_*	Efficiency of refrigeration compressor (%)	*m_ref_*	Refrigerant mass (kg)
*h*_4_	Inlet specific enthalpy of expansion valve (kJ/kg)	T_en_	Ambient temperature (°C)
*h*_5_	outlet specific enthalpy of expansion valve (kJ/kg)	T_cold_	Refrigeration temperature (°C)
*h*_6_	outlet specific enthalpy of evaporator (kJ/kg)	*T_c_*	Condensation temperature (°C)
*q_cold_*	refrigerating capacity per unit mass of refrigerant (kJ/kg)	*T_e_*	Evaporation temperature (°C)
*COP*	Coefficient of performance	*T_op_*	Operating temperature of the precooling air compressor system (°C)
*Ex*	Exergy at the state point (kJ)	*h*_0_	Enthalpy of air at 293.15 K and 101.3 kPa (kJ/kg)

## Appendix A

This paper has conducted a preliminary study on the precooling technology in the energy saving of the air compressor system, and there are still many areas to be improved in the follow-up.

### Appendix A.1. Influences of Humidity on Cooling Energy

When the humidity is too high, the cooling capacity consumed by water vapor will increase the energy consumption of the precooling equipment to a certain extent. When *T_en_* = 20 °C, *P_en_* = 101.3 kPa, *T_cold_* = 5 °C, the air relative humidity changes from 30% to 65%, proportion of air and water vapor cooling energy consumption is shown in Figure A1. Subsequent analysis should be carried out in combination with regional temperature and humidity in order to provide a better reference for practical engineering.

### Appendix A.2. A Pilot Project for Economics Analysis

As for the economy of the precooling air compressor system, tests have been carried out in Binzhou, Shandong Province, China. The rated power of the operating air compressor is 800 kW, and the rated gas production is 9600 m^3^/h. The life cycle of the system is 15 years. The inlet temperature changes periodically with the current weather and temperature. The optimal operating temperature is calculated according to the monthly average temperature (about −1.04~26.25 °C) and monthly average relative humidity (about 45%~70%), and then the *COP* (about 2.25~4.24) under the corresponding working conditions. The average monthly pneumatic-electrical ratio increase is about 4.69%. The economy of the system is analyzed by the method of the whole life cycle cost of the system. On the basis of the technical feasibility of this paper, combined with the actual situation of the application scenario, according to the environmental conditions, timely adjust the working condition of the pre-cooling module, so as to keep the system in the optimal working condition, the system unit cubic gas production cost can be better optimized. Partial results are shown in the table below. Further detailed discussion on system economy is required.

**Table A1:** Related economic parameters.

Relevant Parameter	Traditional	Precooling
Initial outlay *C_Inv_* (million yuan)	1.265	1.265 + 0.4536
Operating cost *C_O_* (million yuan)	43.745856	46.7769
Maintenance cost *C_M_* (million yuan)	0.4635	0.5325
Labor cost *C_Labor_* (million yuan)	1.8	1.8
*LCC* (million yuan)	47.274356	50.8280
Total gas production during life cycle *V_LCC_* (m^3^)	792,025,752.8	881,320,668.9
Unit cubic cost *C_unit_* (yuan /m³)	0.059687903	0.057672584
Percentage decrease in unit cubic cost	(0.059687903 − 0.057672584)/0.059687903% ≈ 3.38%	

Calculation details and instructions:

(1) Initial outlay *C_Inv_*

(A1) $$C_{Inv}=C_{e,Com}+C_{e,Cold}+C_{e,Other}$$

*C_e,Com_* Air compressor acquisition and placement costs;

*C_e,Cold_* Refrigeration equipment acquisition and placement costs;

*C_e,Ohter_* Auxiliary equipment (pipes, valves, etc.) procurement and installation costs (Accounting for 15% of equipment acquisition investment [25]).

(2) Operating cost *C_O_*

(A2)
$$C_{O}=e\cdot t\cdot \left(W_{com}+W_{cold}\right)$$

*e* stands for unit electricity charge;

*t* represents the running time of air compressor system;

*W_com_* represents the sum of the power of each component of the air compressor system;

*W_cold_* indicates the total power of all components in the cooling system (Changes with the weather conditions);

(3) Maintenance cost *C_M_*

(A3)
$$C_{M}=\sum N_{rep}\cdot C_{up}$$

*N_rep_* indicates the number of system consumables (parts) replacement (maintenance);

*C_up_* represents the unit price of consumables (parts) replacement (maintenance);

(4) Labor cost *C_Labor_*

(A4)
$$C_{Labor}=N_{month}\cdot N_{p}\cdot C_{wage}$$

*N_month_* indicates the month of the operation cycle of the air compressor system;

*N_p_* indicates the number of people required to run the system;

*C_wage_* represents the monthly salary of each person in the system location;

(5) *LCC* of the system

(A5)
$$LCC=C_{Inv}+C_{O}+C_{M}+C_{Labor}$$

(6) Total gas production during life cycle *V_LCC_*
  (A6) $$V_{LCC}=t_{year}\cdot V_{year}$$

*t_year_* indicates the full life cycle of the system;

*V_year_* represents the annual gas output of the system;

(7) Unit cubic cost *C_unit_*

(A7)
$$C_{unit}=LCC/V_{LCC}$$

### Appendix A.3. Uncertainty Analysis of the Deviation from Adiabatic Condition

Polytropic index *n* is introduced to express the degree of deviation from adiabatic condition for uncertainty analysis. For industrial air compressors, the polytropic index *n* ranges from 0.9 k to 0.98 k. Variation of the pneumatic-electrical ratio is calculated when *n* is 1.4 and 1.3 respactively to investigate the uncertainty/error caused by veriation of *n*. Energy of air compressor is calculated with following formula,

(A8) $$W_{com}=p_{in}V_{in}\cdot \frac{r^{\frac{n-1}{n}}-1}{\left(n-1\right)/n}/\eta_{com}$$

In this uncertainty analysis, the ambient temperature *T_en_* is set to 20 °C. The intake pressure of air compressors is 101.3 kPa. The pressure ratio *r* is 7.5.

**Table A2:** Uncertainty analysis.

Parameter		Adiabatic Index *k* = 1.4	Polytropic Index *n* = 1.3
Traditional air compressor	*T_in_* (K)	293.15	293.15
	*V_in_* (m^3^)	1	1
	*V*_0_ (m^3^)	1	1
	*W_com_* (kJ)	324.66	305.72
	*δ* (m^3^/kW·h)	11.0884	11.7756
Precooling air compressor	*T_in_* (K)	259.75	259.75
	*V_in_* (m^3^)	1	1
	*V*_0_ (m^3^)	1.129	1.129
	*W_com_* (kJ)	324.66	305.72
	*W_cold_* (kJ)	20.23	20.23
	*δ*′ (m^3^/kW·h)	11.7850	12.4701
Percentage increase in pneumatic-electrical ratio (%)		6.282	5.893
Percentage difference of change rate of pneumatic-electrical ratio		(6.282 − 5.893)/6.282 ≈ 6.192%	

As can be seen from the above table, when the polytrophic index *n* changes from 1.4 to 1.3, the percentage increase in pneumatic-electrical ratio changes from 6.282% to 5.893%, thus 6.282 × (1 − 0.06192)%. Uncertainty of the percentage increase in pneumatic-electrical ratio is 6.192%. Accordingly, the pneumatic-electrical ratio can still be improved by the pre-cooling module when n varies in this range.

### Appendix A.4. Absorption Refrigeration for Waste Heat Recovery and Precooling

In the application of industrial air compressors, up to 80% of the electric energy will be converted into heat. As well, through reasonable design, 50–90% of the heat can be recycled. Applying the absorption refrigeration system to recycle the waste heat and realize the precooling of the air compressor will a good method for further improvement of the efficiency in our future work. Waste heat recovery and precooling are two different concepts to improve the efficiency of a compressor. These two concepts can be combined by absorption refrigeration which recovers waste heat from exhaust air for refrigerating and precooling air compressor. The technical feasibility and economy of the energy-saving scheme need to be considered comprehensively in our future work.
